# The impact of mental health on outcome after anterior cervical discectomy: cohort study assessing the influence of mental health using predictive modelling

**DOI:** 10.1007/s00701-022-05362-z

**Published:** 2022-09-16

**Authors:** Caroline M. W. Goedmakers, Ilse van Beelen, Floor Komen, Erik W. van Zwet, Wilco C. Peul, Mark P. Arts, Carmen L. A. Vleggeert-Lankamp

**Affiliations:** 1 Neurosurgical Center Holland, Department of Neurosurgery, Leiden University Medical Center & Haaglanden MC & Haga Teaching Hospital, Albinusdreef 2, Leiden, 2300 RC the Netherlands; 2 Computational Neuroscience Outcomes Center (CNOC), Department of Neurosurgery, Brigham and Women’s Hospital, Harvard Medical School, Boston, MA USA; 3 Department of Biomedical Data Sciences, Leiden University Medical Center, Leiden, the Netherlands; 4 Department of Neurosurgery, Spaarne Gasthuis, Haarlem, Hoofddorp the Netherlands

**Keywords:** Cervical discectomy, Mental health, Anxiety, Depression, Outcome prediction, Cervical radiculopathy, Neck disability index, Radicular pain

## Abstract

**Background:**

Depression and anxiety are common mental disorders among patients with chronic pain. It is hypothesised that patients suffering from these disorders benefit less from cervical spine surgery than mentally healthy patients. Therefore, this study aimed to quantify the effect of mental health status on functional outcome after anterior cervical discectomy in a post hoc analysis on RCT data.

**Methods:**

One hundred eight patients from the NECK trial, with radiculopathy due to a one-level herniated disc, underwent anterior cervical discectomy and were included into this analysis. Functional outcome was quantified using the Neck Disability Index (NDI), and mental health status was measured using the Hospital Anxiety and Depression Score (HADS) questionnaire. NDI differences were assessed using generalised estimated equations (GEE), crude means, a predictive linear mixed model (LMM) using baseline scores and over time with an explanatory LMM.

**Results:**

At baseline, 24% and 32% of patients were respectively depressed and anxious and had statistically significant and clinically relevant higher NDI scores during follow-up. However, in those patients in which the HADS returned to normal during follow-up, NDI values decreased comparably to the non-depression or non-anxiety cases. Those patients that demonstrated persisting high HADS values had convincingly worse NDI scores. A predictive LMM showed that combining baseline NDI and HADS scores was highly predictive of NDI during follow-up. The R shiny application enabled the effective, visual communication of results from the predictive LMM.

**Conclusion:**

This study shows that mental health status and disability are strongly associated and provides insight into the size of the effect, as well as a way to use this relation to improve preoperative patient counselling. These findings give rise to the suggestion that incorporating mental health screening in the preoperative assessment of patients could help to adequately manage patients’ expectations for functional recovery.

**Trial registration:**

Dutch Trial Register Number: NTR1289

**Supplementary Information:**

The online version contains supplementary material available at 10.1007/s00701-022-05362-z.

## Introduction

Depression and anxiety are common psychological disorders in patients with pain and chronic diseases. A review in JAMA internal medicine reports a 56% mean prevalence of major depression in patients having pain in orthopaedic or rheumatology disease [4]. At the same time, depressive symptoms are known to influence clinical outcome in patients being treated for pain. Patients suffering from depressive symptoms report more pain, more intense pain, more amplification of pain symptoms and longer duration of pain [6, 14, 25, 26]. Additionally, patients with both conditions have a lower self-perceived recovery rate and are more likely to report persistent pain [19, 20, 26, 27].

In a systematic review on the relation between psychological disorders and spine surgery, it was concluded that this group of patients suffers from higher rates of spinal pain, postoperative complications and worsened functional outcomes [12]. Specifically, in patients with lumbar radiculopathy, it was demonstrated that better mental health at baseline was significantly associated with lower disability after surgery [8]. In the cervical spine, however, the relation between mental health and functional outcome after surgery is less well investigated. One study showed statistically significant improvement in postoperative neck pain after 1 year in patients who received treatment for their anxiety compared those who had not [1]. However, longer follow-up on these patients with treated depression demonstrated no significant difference in objective or subjective outcomes up to 24 months after surgery [9]. Another study that described the impact of preoperative depression on outcome after posterior cervical fusion found that depressed patients reported less improvement in postoperative quality of life [2], but this could not be confirmed in a later study [21]. The true size of the effect of mental health on functional outcome after cervical spine surgery thus remains to be unknown. Ultimately, not just the effect size of mental health on functional outcome is of interest but also how the association can be used to effectively counsel patients preoperatively.

Therefore, in this study, the relation between mental health and functional outcome after anterior cervical spine surgery was prospectively studied based on randomised controlled trial (RCT) data. Firstly, the effect size of mental health status on functional outcome was quantified. Secondly, a prediction model was developed and implemented in an application to improve preoperative patient counselling in clinical practice.

## Materials and methods

### Design

In this study, a post hoc analysis was performed on data collected as part of the NECK trial, a prospective, double-blinded multicentre RCT conducted among patients with cervical radiculopathy due to single-level disc herniation. Patients were randomly assigned into three groups: anterior cervical discectomy with arthroplasty (ACDA), anterior cervical discectomy and fusion (ACDF) and anterior cervical discectomy (ACD) alone. Details about the protocol, inclusion criteria, sample size calculations, methods and outcomes of this trial have been previously published [23, 28]. The trial showed small, non-significant and not clinically relevant differences in clinical outcome between the three treatment groups after 2 years. Therefore, all patients from the NECK trial were analysed as one cohort in this study.

### Outcome measures

Data was prospectively collected. The primary clinical outcome measure used in the NECK trial was the Neck Disability Index (NDI). To assess mental health status, the Hospital Anxiety and Depression Scale (HADS) was used. Data on the HADS scores have not previously been published.

The HADS is a patient-reported questionnaire to screen for generalised anxiety disorder (GAD) and depression. The questionnaire consists of 14 items; half of these focus on depression and the other half on anxiety. The HADS score classifies patients into three categories: cases (11–21 points), doubtful cases (8–10 points) or non-cases (0–7 points) for GAD and depression separately [11, 16]. In addition to the inclusion criteria used in the NECK trial, patients needed to have baseline HADS measurement in order to be included into this analysis.

The NDI was used to measure functional outcome. The NDI is a 10-item questionnaire on three different aspects: pain intensity, daily work-related activities and non-work related activities. The total score ranges from 0 (best score) to 50 (worst score) and was converted to a 100-point scale. The NDI is a modification of the Oswestry Low Back Pain Questionnaire and has been shown to be reliable and valid for patients with cervical pathology [18, 22, 24]. Patients were asked to fill out both HADS and NDI questionnaires at baseline, 1 and 2 years after surgery.

### Statistical analysis

The statistical analysis is performed with R version 3.6.0 and RStudio version 1.2.1335. All code is shared in an online, open data repository (Appendix A).

#### Statistics using HADS baseline score

Patient demographics were analysed grouped upon baseline HADS scores (cases, doubtful cases and non-cases) and tested using the chi-squared test for categorical values, the ANOVA tests for parametric numerical variables and the Kruskal–Wallis tests for nonparametric numerical variables. Data was analysed separately for HADS anxiety and HADS depression.

To study how the NDI scores developed over time for each baseline HADS group, generalised estimating equations (GEE) were used. Analysing repeated measurements using GEE allows for estimates of the outcome, based on variation within individuals. The multiple measurements can control for the time invariant and unobservable differences between individuals. In this model, the follow-up moment, the HADS category and the individual patient numbers were used to explain the dependent variable (NDI).

To study how baseline HADS and baseline NDI scores can be used to predict NDI scores 1 and 2 years after surgery, a predictive linear mixed model (LMM) was developed. NDI at baseline and HADS at baseline are centred at its mean, which improves interpretability of the intercept. Part of the within-group variance can be accounted for by adding a random intercept in the LMM. The intraclass correlation (ICC) was calculated to quantify the amount of within-group variance that the random intercept can explain.

The predictive ability of the model is tested using two different methods: predictions for four specific patients and cross-validation (CV). An R shiny application will be developed to implement and visualise the results from the predictive LMM.

#### Statistics using HADS over time

As HADS scores can change over time, the relation between change in HADS was studied in relation to change in NDI scores. Firstly, the Pearson correlation coefficient was calculated between decrease in HADS depression, HADS anxiety and NDI with corresponding *P*-values. Secondly, patients were divided into four groups, based on their change in HADS scores (delta HADS) to study how NDI change related to HADS group change. Patients were categorised into either (1) (doubtful) case at baseline and no (doubtful) case after 2 years, (2) (doubtful) case at baseline and (doubtful) case after 2 years, (3) no (doubtful) case at baseline and (doubtful) case after 2 years and (4) no (doubtful) case at baseline and no (doubtful) case after 2 years. There was no distinction made between anxiety and depression in delta HADS groups.

Additionally, in order to study HADS as a continuous variable, an explanatory LMM was used to analyse the dependent variable NDI over time, in relation to total HADS and follow-up time as independent variables. HADS is centred at its mean, for the same reasons as mentioned for the predictive LMM.

## Results

### HADS at baseline

#### Baseline characteristics

**Fig. 1:**
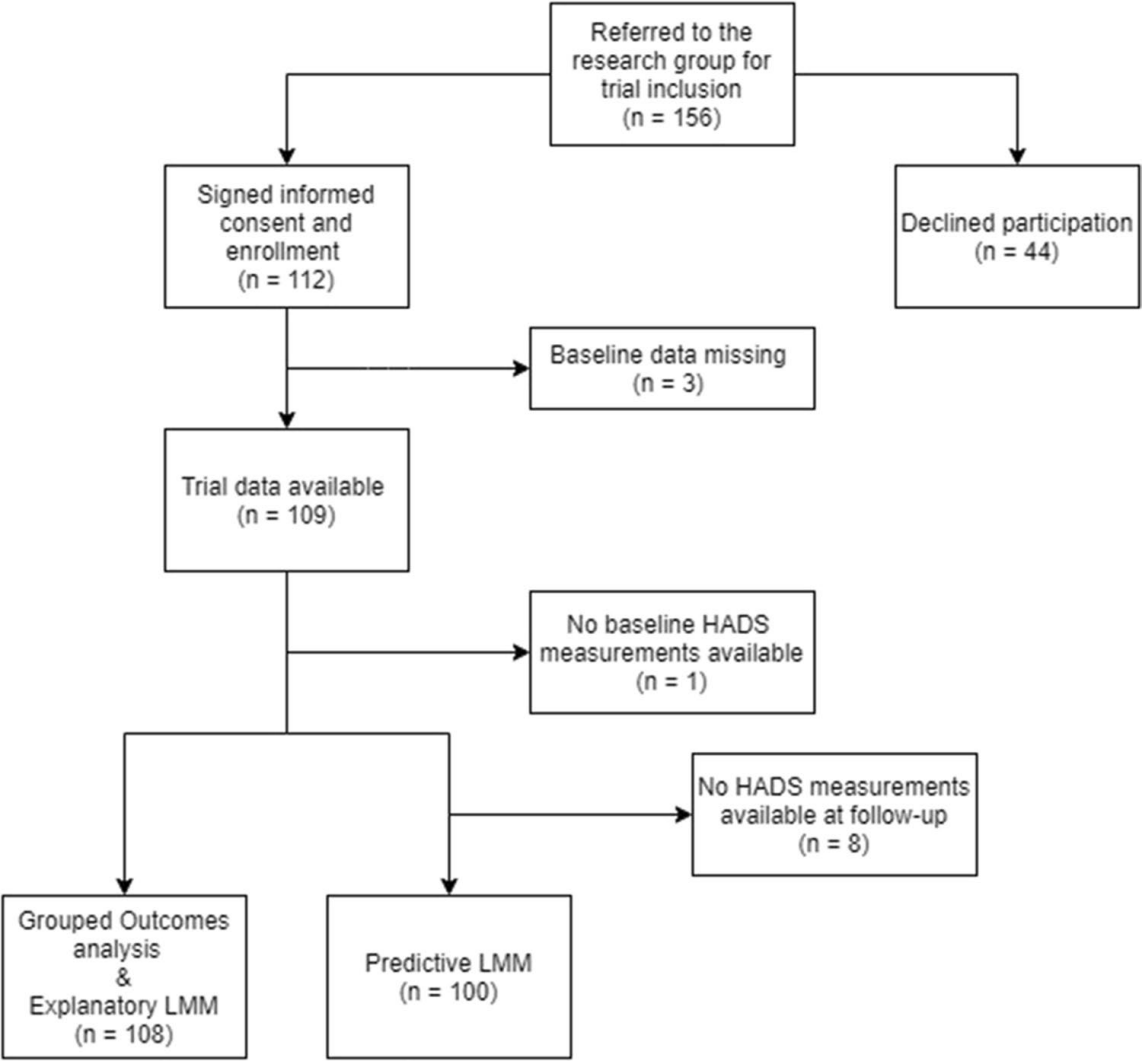
Flow chart illustrating the inclusion process

One hundred eight patients were included in this analysis; patients needed to have at least completed NDI and HADS measurements at baseline (Fig. 1). Patients were on average 46.8 ± 7.9 years old, 52% was female, BMI was 26.6 ± 4.3, NDI score was 44.4 ± 15.4, and the median duration of complaints was 26.0 (IQR 39) weeks. At baseline, 12 patients (11%) were classified as ‘depression cases’, and 14 patients (13%) were classified as ‘GAD cases’. Ten of those patients were classified as both depression and GAD cases.

NDI at baseline was significantly higher, indicating higher disability, for both the depression and the anxiety cases as compared to the doubtful and the non-cases (*p* < 0.001; Table 1). There were no other statistically significant differences between cases, doubtful cases and non-cases at baseline when grouping on either baseline HADS depression or anxiety scores.

**Table 1:** Patient characteristics per baseline HADS group and total

	Depression			Anxiety			Total
	Non-cases	Doubtful cases	Cases	Non-cases	Doubtful cases	Cases	
Number (*n*)	82	14	12	73	21	14	108
Age (yr)	46.6 ± 8.4	45.6 ± 4.4	48.8 ± 8.1	46.6 ± 8.2	47.6 ± 6.5	46.1 ± 8.8	46.8 ± 7.9
Sex (F/M)	43/39	5/9	8/4	38/35	10/11	8/6	56/52
BMI	26.6 ± 4.3	26.6 ± 3.9	27.1 ± 5.0	26.7 ± 4.3	25.6 ± 4.7	28.0 ± 3.7	26.6 ± 4.3
NDI	40.7 ± 13.3*	45.3 ± 11.8*	68.7 ± 9.2*	40.1 ± 13.5*	45.9 ± 9.9*	64.4 ± 15.3*	44.4 ± 15.4
Duration complaints (weeks)	26.0 (39)	32.5 (36)	24.0 (30)	26.0 (39)	26.0 (35)	19.5 (18)	26.0 (39)

^*^Indicates a significant difference between the groups. All other characteristics were similar between groups without reaching statistical significance. Parametric numerical data was represented by mean value ± standard deviation (SD) and nonparametric data as median (interquartile range)

#### Evaluation of NDI in patients grouped by baseline HADS score

Mean values for NDI decreased significantly from 41–47 points at baseline to 18–21 points at 1 year and 19–20 points at 2-year follow-up in all three treatment groups (*p* < 0.001), without significant differences between the three treatment arms (ACD, ACDF or ACDA) [23].

**Fig. 2:**
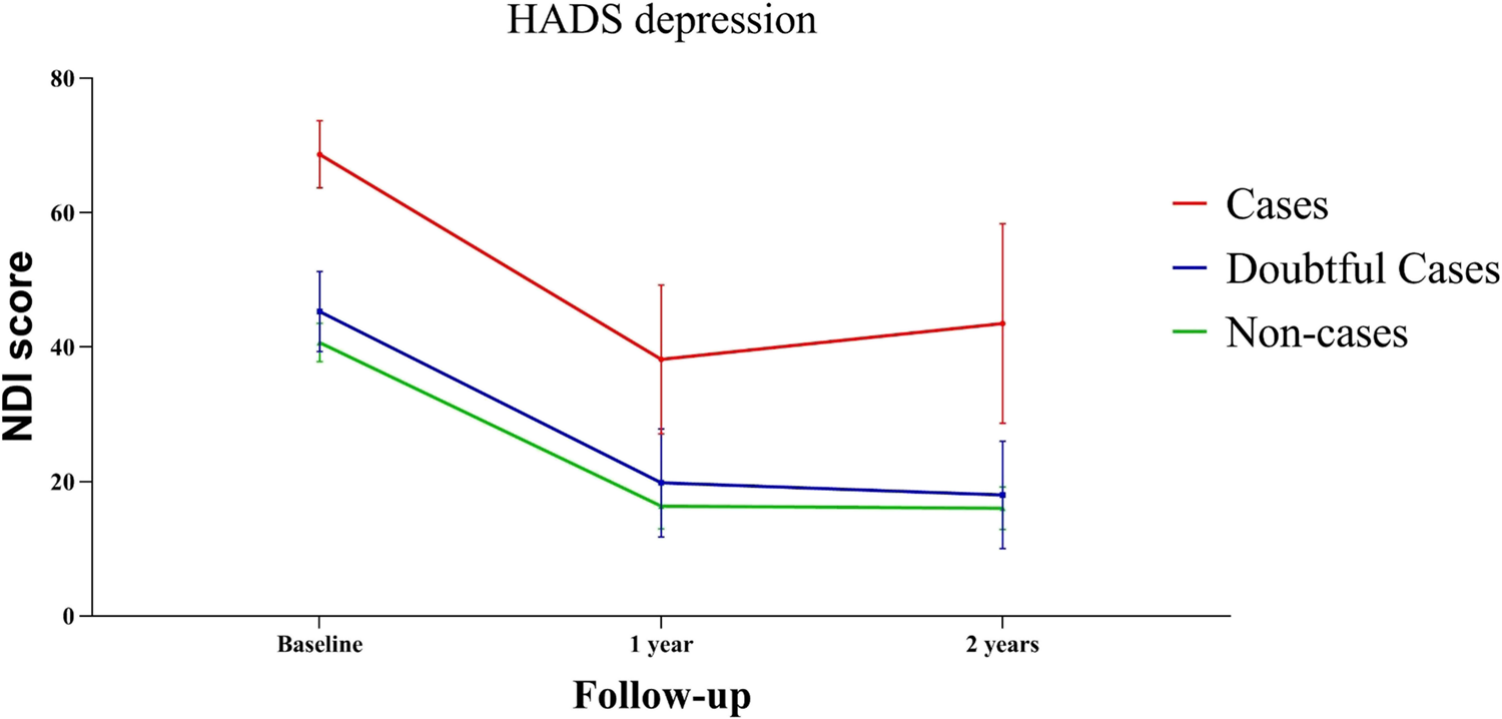
NDI during follow-up for each HADS depression group. HADS depression groups are based on the baseline HADS scores reported

Grouped by the HADS depression score at baseline, depression cases report a marginal mean NDI that was more than doubled at baseline (28.0 points higher) and 2 years after surgery (27.5 points higher) when compared to non-cases (Table 2 and Fig. 2). The difference is not only statically significant (*p* < 0.001) but more importantly clinically relevant, as the minimal clinically important difference (MCID) for NDI is estimated at 20 points [3, 13, 15, 17]. Likewise, depression cases showed comparably higher scores in comparison to doubtful depression cases at all timepoints, while there were small differences in NDI between doubtful and non-cases.

**Table 2:** The difference in estimated marginal mean NDI between HADS depression groups calculated using generalised estimated equations

Follow-up	Comparison between depression status		Mean NDI difference	*SE*	Lower bound 95% CI	Upper bound 95% CI	*P*-value
Baseline	Cases	Non-cases	28.0	2.9	22.2	33.8	< 0.001
	Cases	Doubtful cases	23.4	4.0	15.6	31.1	< 0.001
	Doubtful cases	Non-cases	4.6	3.4	− 2.0	11.2	0.170
1 year postoperative	Cases	Non-cases	21.8	5.9	10.3	33.4	< 0.001
	Cases	Doubtful cases	18.4	7.0	4.7	32.0	0.009
	Doubtful cases	Non-cases	3.5	4.4	− 5.2	12.2	0.434
2 years postoperative	Cases	Non-cases	27.5	7.7	12.3	42.7	< 0.001
	Cases	Doubtful cases	25.5	8.6	8.7	42.4	0.003
	Doubtful cases	Non-cases	2.0	4.4	− 6.6	10.6	0.651

^*^Groups based on HADS anxiety scores at baseline

^**^HADS Hospital Anxiety and Depression Scale, *SE* standard error, *NDI* Neck Disability Index, *CI* confidence interval

**Fig. 3:**
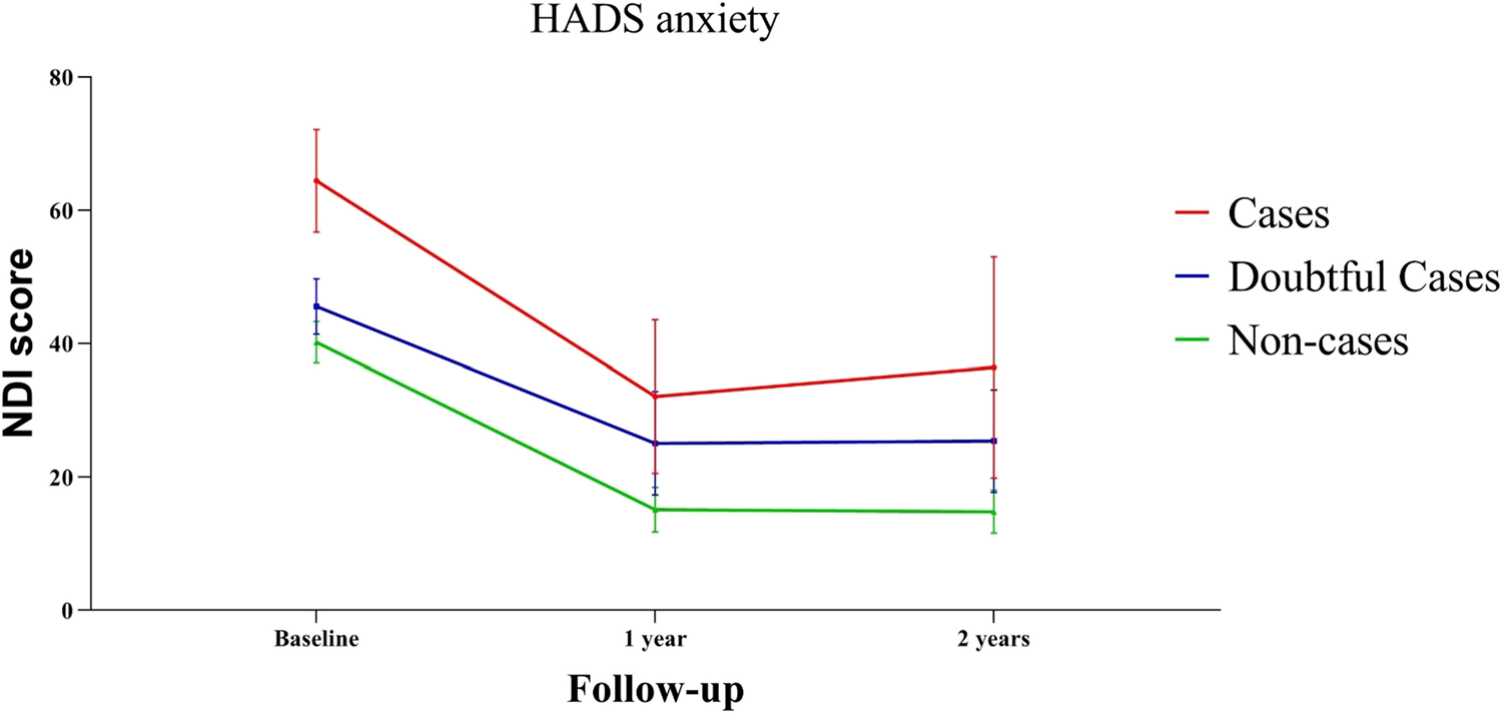
NDI during follow-up for each HADS anxiety group. HADS anxiety groups are based on the baseline HADS scores reported

Grouped according to baseline HADS anxiety score, anxiety cases report a 24.2, 17.0 and 21.6 points higher NDI score when compared to non-cases, respectively, at baseline, 1 year after surgery and 2 years after surgery (Table 3 and Fig. 3). The difference is statistically significant (*p* < 0.001, *p* = 0.006, *p* = 0.012) and exceeds the MCID. At baseline, the difference between the anxiety cases and doubtful cases is 18.9 (*p* < 0.001); however, 1 and 2 years postoperatively, the differences between cases and doubtful are similar to those between the doubtful cases and non-cases (Table 3 and Fig. 3).

**Table 3:** The difference in estimated marginal mean NDI between HADS anxiety groups calculated using generalised estimated equations

Follow-up	Comparison between anxiety status		Mean NDI difference	*SE*	Lower bound 95% CI	Upper bound 95% CI	*P*-value
Baseline	Cases	Non-cases	24.2	4.2	15.9	32.5	< 0.001
	Cases	Doubtful cases	18.9	4.5	10.1	27.6	< 0.001
	Doubtful cases	Non-cases	5.4	2.6	0.2	10.6	0.042
1 year postoperative	Cases	Non-cases	17.0	6.1	5.0	28.9	0.006
	Cases	Doubtful cases	7.0	7.1	− 6.9	20.9	0.323
	Doubtful cases	Non-cases	10.0	4.3	1.5	18.4	0.021
2 years postoperative	Cases	Non-cases	21.6	8.6	4.7	38.5	0.012
	Cases	Doubtful cases	11.0	9.3	− 7.2	29.3	0.237
	Doubtful cases	Non-cases	10.6	4.3	2.3	19.0	0.013

^*^Groups based on HADS anxiety scores at baseline

^**^HADS Hospital Anxiety and Depression Scale, *SE* standard error, *NDI* Neck Disability Index, *CI* confidence interval

#### Predicting NDI based on baseline HADS score

A predictive LMM was used to predict the NDI values after 52 and 104 weeks, based on the baseline NDI and HADS values. The estimates of the beta coefficients were respectively 0.37 (*p* < 0.001) and 0.73 (*p* < 0.001) (Table 4). The beta coefficient calculated for time in weeks was not significant (*p* = 0.75), most likely due to the small average differences in NDI scores between the two timepoints.

**Table 4:** Coefficients of the predictive linear mixed effects model with its corresponding standard error (SE), degrees of freedom (DF), *t*-value and *P*-value. Given the mean NDI of 44 and the mean HADS of 11, if a patient has an NDI of 54 (deviation of 10 with the mean NDI) and a HADS of 10 (deviation of − 1 with the mean HADS) at baseline, the NDI after 2 years will be predicted as 18.98 + 0.37 ∙ 10 + 0.73 ∙ − 1 + 0.0081 ∙ 104 = 22.79

	Coefficients	*SE*	*DF*	*t*-value	*P*-value
Intercept	18.98	2.43	161.49	7.82	< 0.001
Deviation NDI at baseline	0.37	0.11	99.89	3.29	< 0.001
Deviation HADS at baseline	0.73	0.24	98.48	3.07	< 0.001
Time in weeks	0.0081	0.026	91.89	0.32	0.75
Time with symptoms	− 0.18	0.27	16.90	− 0.65	0.5229
VAS neck pain	0.06	0.07	96.42	0.92	0.3590
VAS arm pain	0.06	0.053	96.73	1.15	0.2550
Osteofyte/spondylosis	− 5.93	4.36	56.35	− 1.36	0.1789
Positive family history for neck problems	− 2.81	6.35	96.72	− 0.44	0.6591
Age	0.14	0.18	102.44	0.78	0.4364
Gender	2.53	2.83	98.85	0.89	0.3744
Smoking	0.55	2.90	99.70	0.19	0.8494
Alcohol use	1.19	3.08	98.96	0.39	0.6999
BMI	− 0.40	0.33	94.61	− 1.23	0.2223
Disc height at index	− 1.35	1.12	84.77	− 1.21	0.2312

**Fig. 4:**
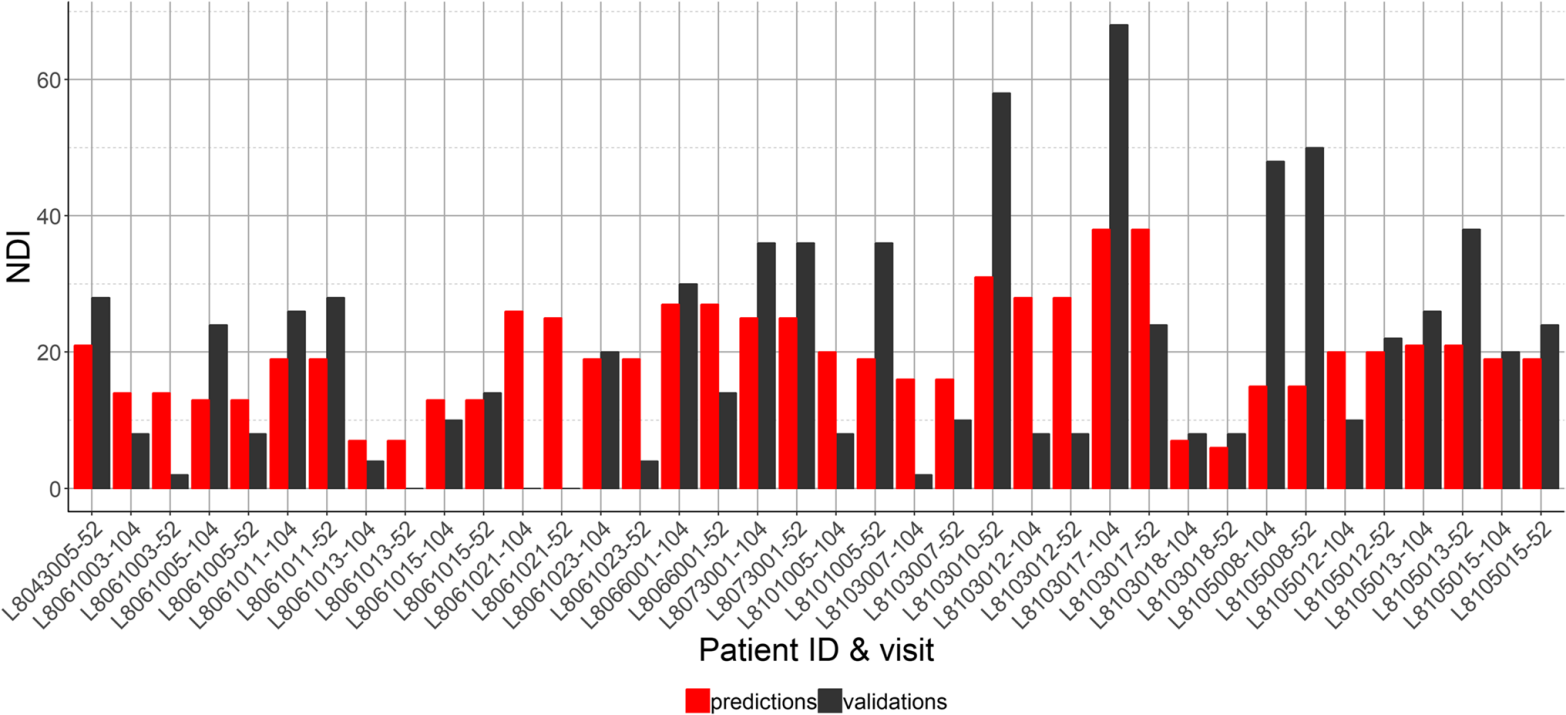
5-fold cross-validation (CV) with the predictions (in red) and actual NDI (in dark grey) of one fold. The *x*-axis visualises the specific anonymized patient ID combined with a visit (52 or 104 weeks). For CV, the data is randomly divided into 5 parts, where 4 parts will function as the training data and the 5th part as the test data. The procedure is repeated 5 times. The lower the root mean square error (RMSE), the better the predictive ability of the model, and the RMSE can also be interpreted as the average amount of NDI points that the model predicts less or more than the actual NDI

**Fig. 5:**
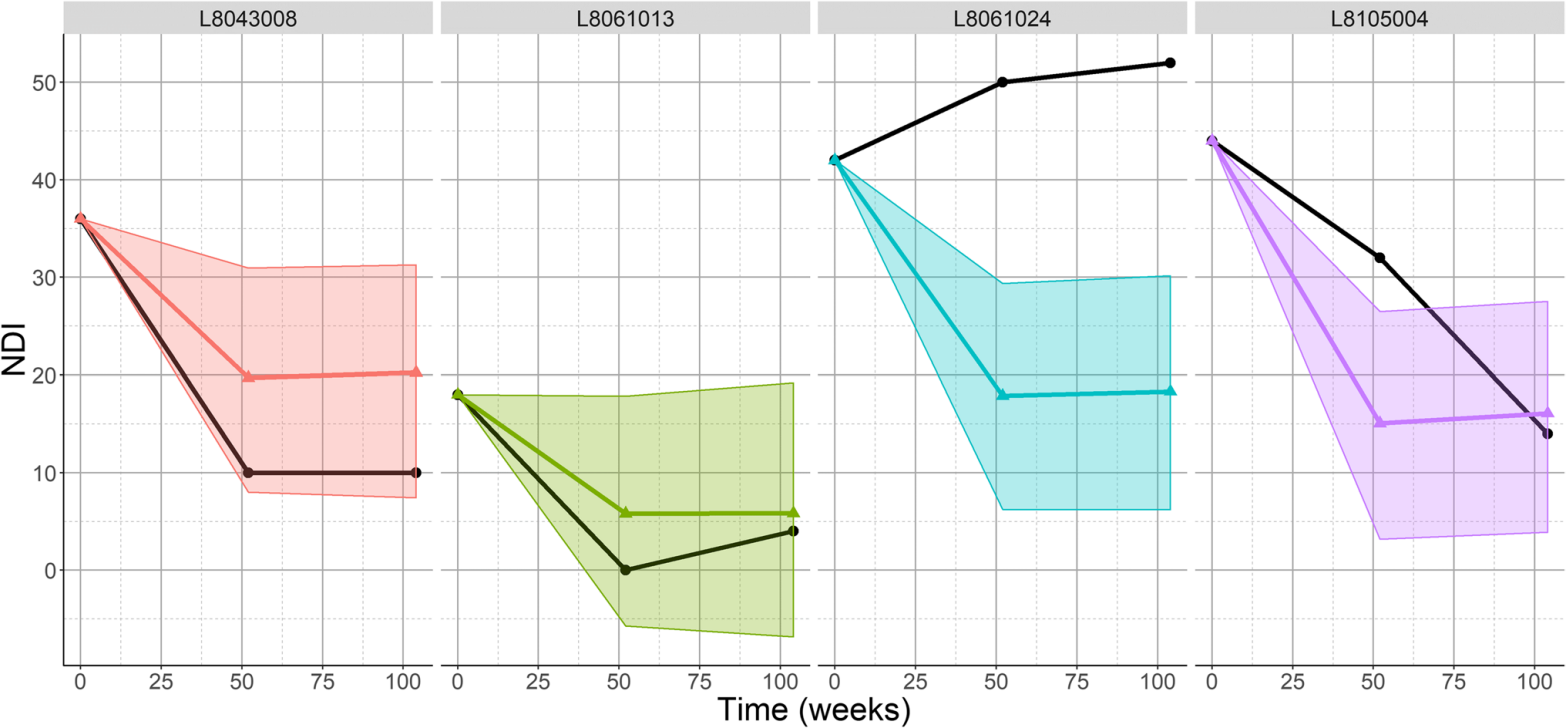
Predicted NDI values on weeks 52 and 104 and their confidence intervals for four individual patients are shown in colour. The black lines are the true NDI values. In the patient-specific predictions, the training data is created by removing one patient from the dataset. The predictive LMM is trained on this training data and predicts for the left out patient. This procedure is repeated four times, for four different patients

The *RMSE* ± *SD* is after a fivefold CV calculated to be 14.5 ± 1.7 after 52 weeks and 15.8 ± 1.6 after 104 weeks and did not show a clear pattern of model under or overprediction (Fig. 4). Comparing individual predictions shows that predictions for patients that have much higher than average NDI, at either 52 weeks or 104 weeks, are not within the confidence interval of the predicted value (Fig. 5).

The ICC was 0.75. NDI and HADS at baseline are therefore highly predictive for NDI at 52 and 104 weeks. To investigate whether the remaining 0.25 variance could be further explained, other covariates were added to the predictive LMM. Weight, beta coefficient − 0.19 (*p* = 0.0313), and height, beta coefficient − 0.32 (*p* = 0.0193), were both statistically significant (Table 4), while BMI was not (*p* = 0.2223). Time with symptoms, VAS neck pain, VAS arm pain, osteophyte/spondylosis, positive family history, age, gender, smoking, alcohol use and disc height were also not statistically significant.

**Fig. 6:**
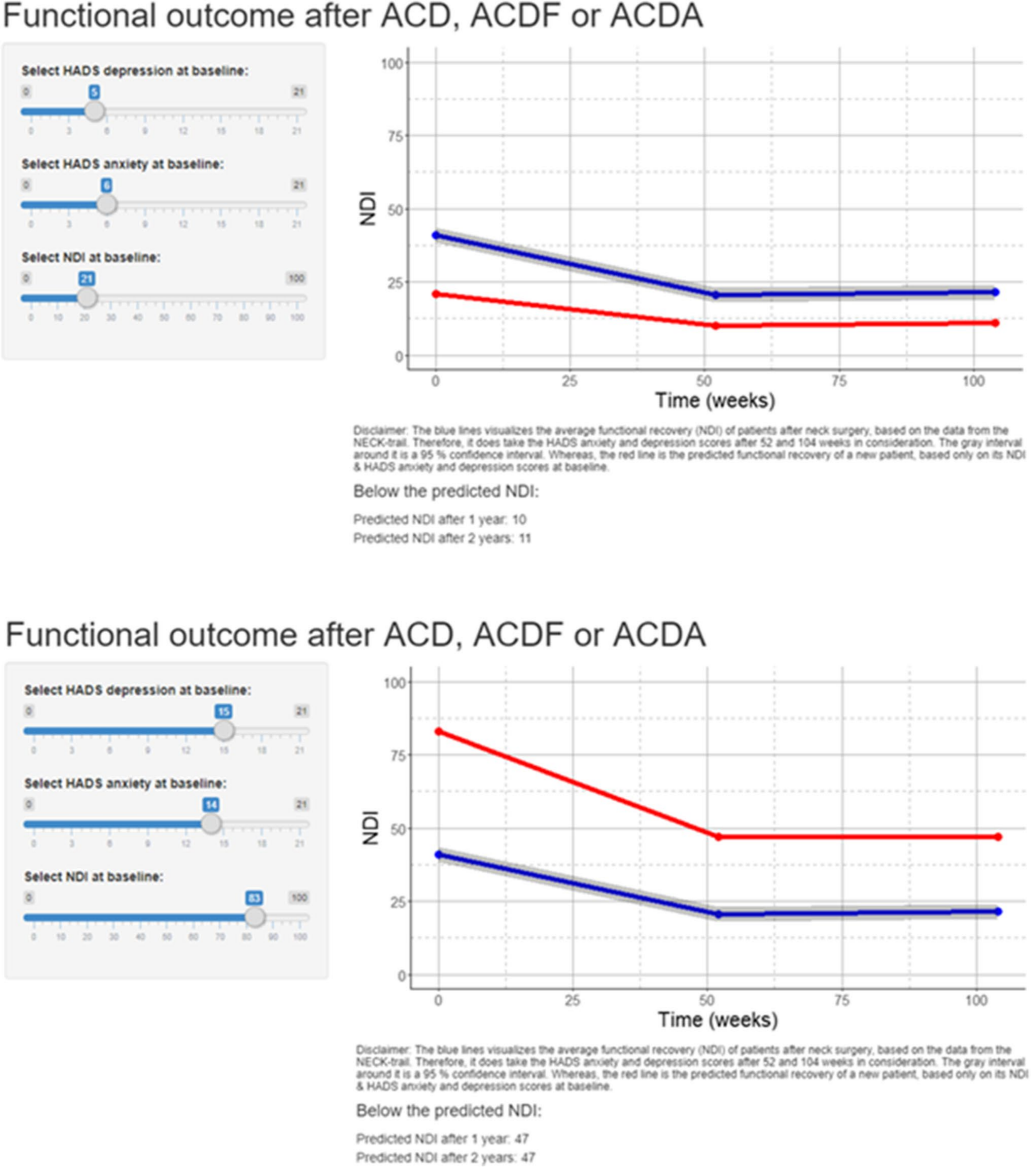
Screenshots of the R shiny application illustrating how it functions. On the left, the input can be given, and on the right, the predicted NDIs will be visualised in the graph (red line). On the left side, the adjustable baseline measurements for NDI, HADS anxiety and HADS depression. On the right, the graph visualising (red line) the predicted NDI on the *y*-axis during the follow-up moment in weeks on the *x*-axis, and the marginal mean NDI (blue line) over time with a 95% confidence interval (in grey). Beneath the graph, the numerical values appear for the predicted NDI at weeks 52 and 104

In order to visualise the results of the predictive LMM, an R shiny application was developed. The application allows the physician to communicate the results of the LMM visually to the patient using a dynamic graph (Fig. 6).

### HADS during follow-up

#### Evaluation of NDI in patients grouped by delta HADS score

**Fig. 7:**
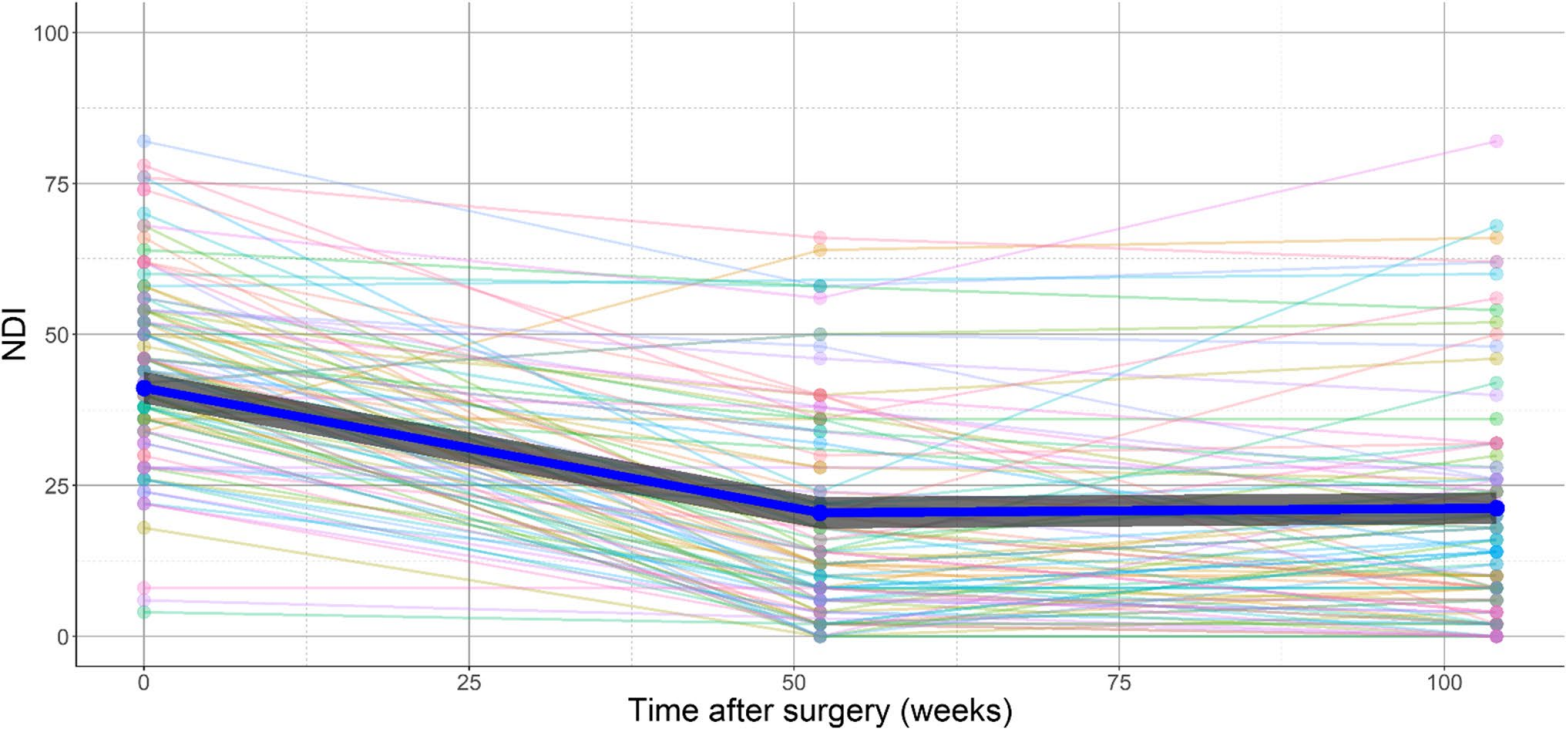
Marginal NDI over time (in blue) with a 95% confidence interval (dark grey). The NDI of individual subjects is plotted in the background

**Fig. 8:**
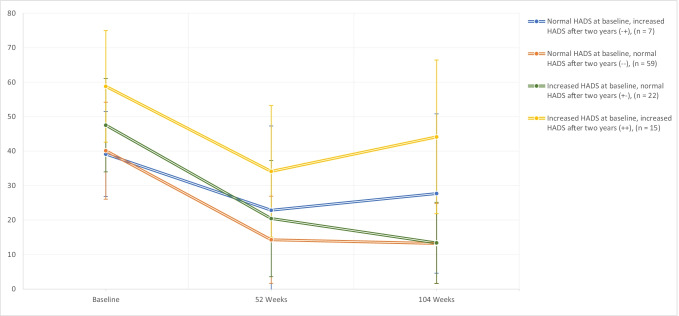
NDI during follow-up for the delta NDI groups. Patients were grouped based on their HADS score over time

Decrease in HADS depression score was correlated to a decrease in NDI, and likewise, the decrease in HADS depression was correlated to a decrease in HADS anxiety (Pearson correlation coefficient 0.544 and 0.552, respectively (*p* < 0.001) (Table 5 and Appendix D)). Furthermore, the decrease in NDI during the first year was comparable in the three groups (cases, doubtful cases and non-cases), and the value of NDI remained stable after 1 year in all groups (based on baseline HADS scores) (Figs. 2, 3, 7). However, HADS scores were measured again after 1 and after 2 years and changed during follow-up in some patients. To evaluate the effect on NDI, patients were additionally differentiated based on their change in HADS (delta HADS) into no (doubtful) case at baseline and (doubtful) case after 2 years (*n* = 7), no (doubtful) case at baseline and no (doubtful) case after 2 years (*n* = 59), (doubtful) case at baseline and no (doubtful) case after 2 years (*n* = 22) or (doubtful) case at baseline and (doubtful) case after 2 years (*n* = 15). Remarkably, NDI changed in the same direction as the HADS score changes (Fig. 8 and Appendix E). Patients with increasing HADS scores report higher NDI scores, an interaction that can also be seen with change in the opposite direction; with a decreasing HADS and lower NDI scores (Fig. 8 and Appendix E).

**Table 5:** Correlations between decrease in HADS depression, HADS anxiety and NDI from baseline to 2 years after surgery

		Decrease in NDI	Decrease in HADS depression score	Decrease in HADS anxiety score
Decrease in NDI	Pearson Correlation coefficient	1	0.544	0.347
	*P*-value		< 0.001	0.001
Decrease in HADS depression score	Pearson Correlation coefficient	0.544	1	0.552
	*P*-value	< 0.001		< 0.001
Decrease in HADS anxiety score	Pearson Correlation coefficient	0.347	0.552	1
	*P*-value	0.001	< 0.001	

^*^Correlation is significant at the 0.01 level (2-tailed)

^**^HADS Hospital Anxiety and Depression Scale, *NDI* Neck Disability Index

#### Explanatory linear mixed effects model

To quantify the effect of HADS on NDI over time, follow-up moment and deviation from the mean HADS were used in the LMM (Appendix F). The intercept of 41.16 can be interpreted as the NDI at baseline for a patient with an average HADS score, with a decrease of 20.68 points in NDI after 1 year compared to baseline or 19.92 after 2 years compared to baseline. All predictors are significant (*p* < 0.001) (Appendix F). Deviation HADS has a beta coefficient of 1.34, meaning that one point increase of HADS compared with the mean HADS results in an NDI increase of 1.34, regardless of time (Table 6). The ICC is 0.24, lower than for the predictive LMM, most likely due to the fact that the NDI at baseline is an outcome in this model and not a predictor, highlighting the previously discussed importance of baseline NDI for accurate estimation of NDI over time.

**Table 6:** Coefficients of explanatory linear mixed effects model with its corresponding standard error (*SE*), degrees of freedom (*DF*), *t*-value and *P*-value. HADS is centred at its mean

	Coefficients	*SE*	*DF*	*t*-value	*P*-value
Intercept	41.16	1.32	187.00	31.25	< 0.001
Time: 52 weeks	− 20.68	1.45	187.00	− 14.31	< 0.001
Time: 104 weeks	− 19.92	1.45	187.00	− 13.75	< 0.001
Deviation HADS	1.34	1.34	187.00	11.67	< 0.001

## Discussion

Patients that were classified as depression or anxiety cases at baseline had statistically significant and clinically relevant higher NDI scores 1 and 2 years after surgery. The crude NDI averages for the delta HADS groups illustrate that also during follow-up NDI changes in the same direction as the HADS group changes. Additionally, the predictive LMM and explanatory LMM enabled the successful analysis of decreased mental health using HADS on a continuous scale, as it relates to functional outcome in NDI respectively over time. Lastly, a method was proposed to effectively communicate the results from the predictive LMM, using an R shiny application.

These results raise the question whether decreased mental health status is either a patient characteristic or comes inherently with experiencing pain and disability from cervical disc disease. Whether disability causes symptoms of decreased mental health or whether decreased mental health causes patients to experience higher disability is not assessed in this study. A combination of both is assumed to be most likely, which would support the recommendation for future research to assess if preoperative treatment of depressive or anxious symptoms could improve functional outcome after surgery, both immediately postoperative as well as long term. This has only been shown in one previous study on a relatively small number of patients that used a pharmaceutical intervention [5]. Other than pharmaceutical intervention, preoperative counselling or cognitive behavioural therapy could be other potentially interesting strategies to investigate.

The use of prospectively collected, high quality data from an RCT is a major strength of this study. This allowed for repeated measurement analysis of the HADS scores and assessment of their predictive value. Furthermore, it provided the opportunity to analyse HADS scores on a continuous scale, rather than dichotomizing results and losing the granular aspect of this outcome scale, as has been done previously [21]. Another strength is the direct implementation of predictive modelling results into an application. Improving presurgical counselling possibilities in this manner is increasingly important with the rise of shared decision-making in the current medical world, for which effective communication of research results is paramount.

However, this study has limitations. A limitation for the external validity of this study is the exclusion of patients with severe mental and psychiatric disorders from the NECK trial. However, it could be argued that excluding severely depressed patients strengthens our conclusions, as the effect was illustrated in patients suffering from relatively ‘mild’ symptoms.

The use of only clinical, without radiological or histological, parameters is another limitation to this study and was illustrated by the percentage of within-group variance that could not be explained. The explanatory LMM showed that using only HADS scores explained 24% (ICC 0.24) of the within-group variance of NDI scores, whereas the predictive LMM, that incorporated baseline NDI score as well, adds another 51% (ICC 0.75). However, there remains to be 25% of variance unexplained, and therefore, we plea to combine different types of outcome parameters, as well as increasing the sample size of analysed patients, in future research in order to achieve higher accuracy in predictions and bring the percentage of unexplained variance down. Radiological imaging data has recently been successfully used in cervical spine disease to predict outcomes with deep learning techniques [10]. Other studies have shown how in sciatica the histopathological parameters in disc tissue, such as different types of pro-inflammatory cytokines, could be associated with worsened pain symptoms [7]. However, the combination of both radiological, clinical and histological parameters could draw an even more complete picture of the patient and can therefore be expected to achieve higher accuracy in predicting outcomes for individual patients.

Moreover, the duration of follow-up is another limitation, as the analysed data was collected at baseline, 1 and 2 years after surgery, but ideally, clinical predictions would be made for long-term outcomes, 5 to 10 years after surgery.

Lastly, in future causality research, determining the direction of the effect between mental health and disability scores after cervical spine surgery should be addressed, as it may provide additional insights on how to manage patients with mental illnesses before and after spine surgery.

## Conclusion

Patients suffering from depression and anxiety before cervical spine surgery demonstrate significantly more neck disability 1 and 2 years after surgery and therefore do not benefit from surgery in the same way other patients do. Additionally, this study demonstrates that if, during follow-up, symptoms of depression and anxiety improve, patients’ functional outcome improves as well. Using predictive modelling, it was additionally shown that mental health can be used to explain and predict the changes in neck disability after surgery. Lastly, an R shiny application was developed to facilitate an easier-to-interpret visual communication of these models to patients during a preoperative clinic visit.

Using applications, like the one designed in this study based on the predictive modelling developed, can aid personalised treatment counselling and is a promising development for future shared decision-making healthcare.

## Supplementary Information

Below is the link to the electronic supplementary material.

Supplementary file1 (DOCX 227 KB)

Supplementary file2 (DOC 91 KB)

## Abbreviations

ACDF: Anterior cervical discectomy and fusion
ACDA: Anterior cervical discectomy and arthroplasty
GEE: Generalised estimated equations
HADS: Hospital Anxiety and Depression Score
LMM: Linear mixed model
NDI: Neck Disability Index
