# Supplementary material for: The impact of mental health on outcome after anterior cervical discectomy: cohort study assessing the influence of mental health using predictive modelling
Source: Acta Neurochir (Wien). 2022 Sep 16;164(11):3035–46. doi: 10.1007/s00701-022-05362-z (PMC9613752; doi:10.1007/s00701-022-05362-z)
Supplement: Supplementary file 1 — Supplementary file1 (DOCX 227 KB) [file 701_2022_5362_MOESM1_ESM.docx]

**Appendices**

A Code

All code can be found on the Github <https://github.com/ilsevb95/Stat_Consulting>

B Model Assumption plots for explanatory LMM, from left to right: residuals plot
 (homogeneity of variance), QQ-plot (normality), Cook's distance (no outliers)


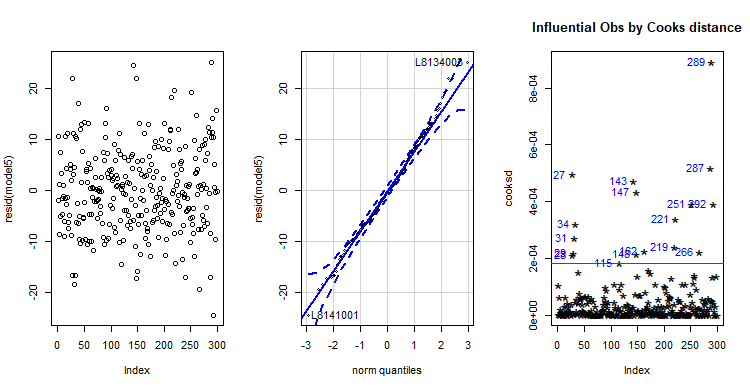


C Histograms of distribution for NDI & HADS total scores


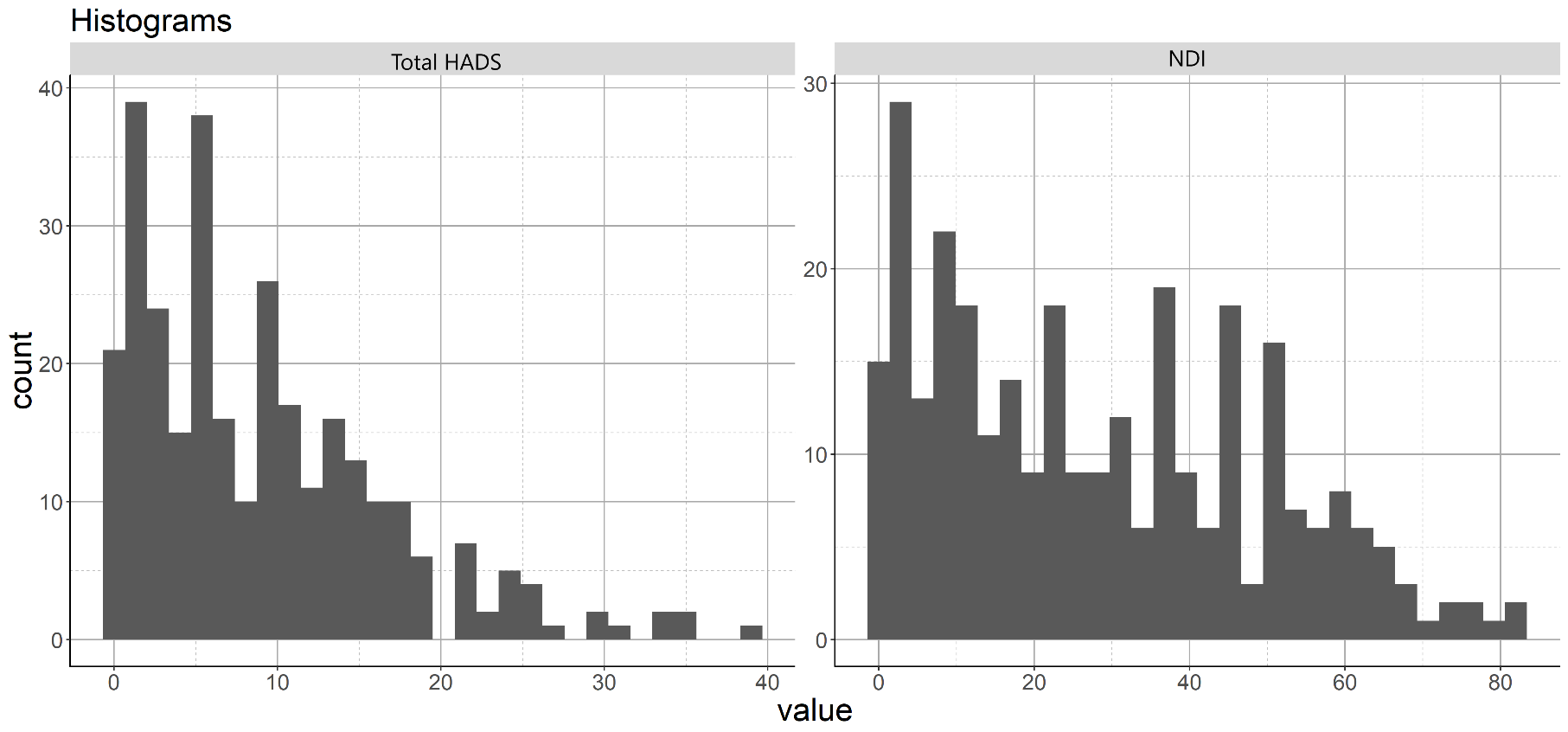


D Correlation plots of NDI at baseline with NDI after 1 and 2 years


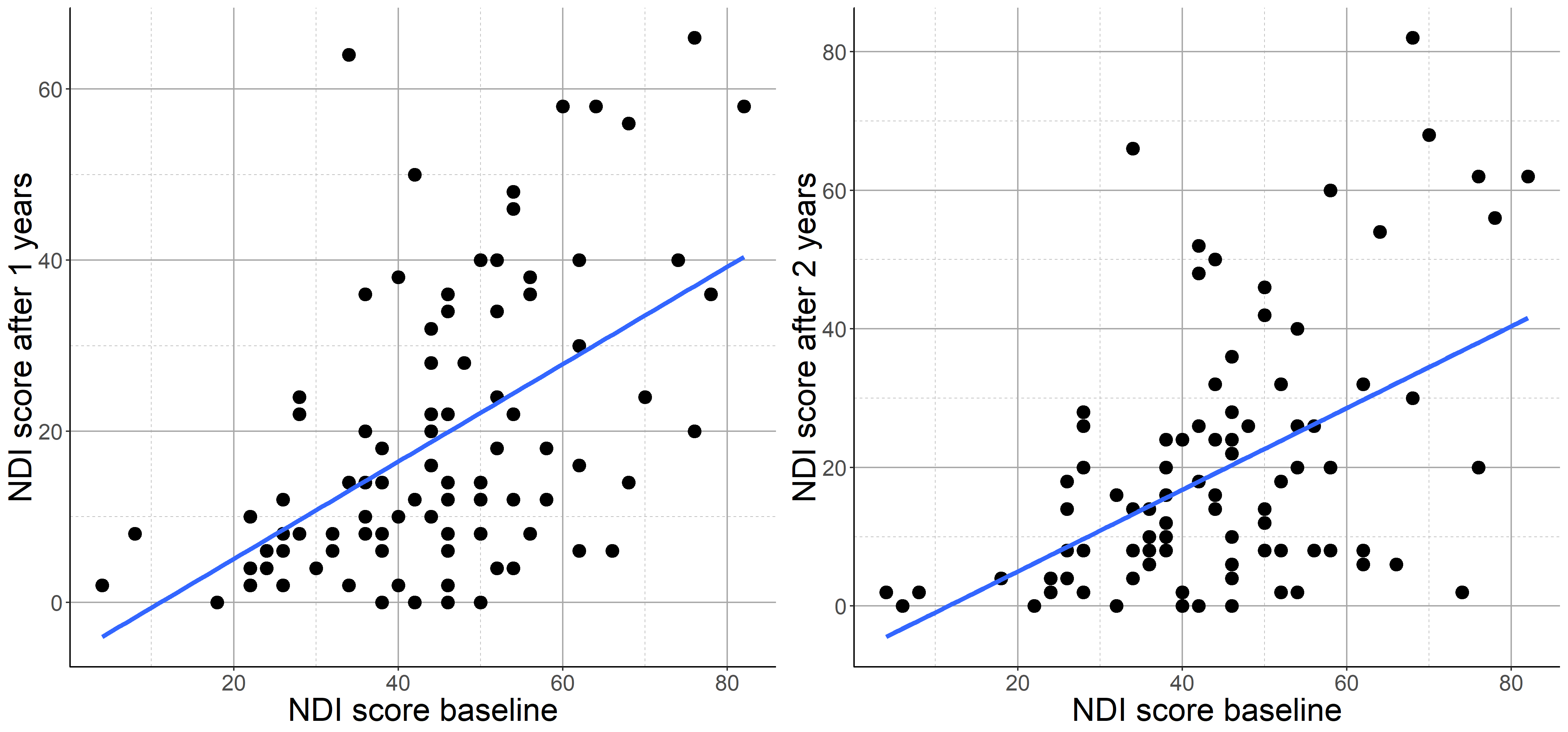


E Crude average NDI with standard deviation per delta HADS group

| **Delta HADS group** | **Baseline** | **52 Weeks** | **104 Weeks** |
| --- | --- | --- | --- |
| No (doubtful) case at baseline,  (doubtful) case after two years | 39.14 ± 12.32 | 22.86 ± 24.46 | 27.71 ± 23.11 |
| No (doubtful) case at baseline,  no (doubtful) case after two years | 40.14 ± 14.07 | 14.30 ± 12.65 | 13.22 ± 11.63 |
| (Doubtful) case at baseline,  no (doubtful) case after two years | 47.55 ± 13.56 | 20.45 ± 16.88 | 13.40 ± 11.77 |
| (Doubtful) case at baseline,  (doubtful) case after two years | 58.80 ± 16.19 | 34.14 ± 19.09 | 44.13 ± 22.29 |
